# The histone deacetylase inhibitor trichostatin A downregulates human *MDR1* (*ABCB1*) gene expression by a transcription-dependent mechanism in a drug-resistant small cell lung carcinoma cell line model

**DOI:** 10.1038/sj.bjc.6603914

**Published:** 2007-07-31

**Authors:** V El-Khoury, G Breuzard, N Fourré, J Dufer

**Affiliations:** 1 Unité MéDIAN-CNRS UMR 6142, IFR 53, Faculté de Pharmacie, Université de Reims Champagne-Ardenne, F-51096 Reims, France

**Keywords:** trichostatin A, epigenetics, drug resistance

## Abstract

Tumour drug-resistant *ABCB1* gene expression is regulated at the chromatin level through epigenetic mechanisms. We examined the effects of the histone deacetylase inhibitor trichostatin A (TSA) on *ABCB1* gene expression in small cell lung carcinoma (SCLC) drug-sensitive (H69WT) or etoposide-resistant (H69VP) cells. We found that TSA induced an increase in *ABCB1* expression in drug-sensitive cells, but strongly decreased it in drug-resistant cells. These up- and downregulations occurred at the transcriptional level. Protein synthesis inhibition reduced these modulations, but did not completely suppress them. Differential temporal patterns of histone acetylation were observed at the *ABCB1* promoter: increase in H4 acetylation in both cell lines, but different H3 acetylation with a progressive increase in H69WT cells but a transient one in H69VP cells. *ABCB1* regulations were not related with the methylation status of the promoter −50GC, −110GC, and Inr sites, and did not result in further changes to these methylation profiles. Trichostatin A treatment did not modify MBD1 binding to the *ABCB1* promoter and similarly increased PCAF binding in both H69 cell lines. Our results suggest that in H69 drug-resistant SCLC cell line TSA induces downregulation of *ABCB1* expression through a transcriptional mechanism, independently of promoter methylation, and MBD1 or PCAF recruitment.

The overexpression of the *ABCB1* (*MDR1*) gene product P-glycoprotein (P-gp) has been identified as an important mediator of multidrug resistance (MDR) to chemotherapeutic agents, which can lead to increased tumour resistance and worse prognosis in cancer patients (Gottesman, 2002; Longley and Johnston, 2005).

Small cell lung carcinoma (SCLC) is usually responsive to chemotherapy, but long-term survival is rare. Several chemotherapeutic agents active in SCLC patients (as doxorubicin, etoposide, or vincristine) are P-gp substrates, raising the question of the impact of *ABCB1* expression in these types of tumours. In patient samples, good correlations have been reported between increased *ABCB1* expression, lack of response to chemotherapy, and shorter survival (Holzmayer *et al*, 1992; Poupon *et al*, 1993; Savaraj *et al*, 1997), suggesting that *ABCB1* gene expression may play a significant role in drug resistance in SCLC.

Considerable progress has been made towards the definition of the regulatory mechanisms that control the expression of *ABCB1* gene in tumour cells (for review, see Labialle *et al*, 2002; Scotto, 2003). The *ABCB1* promoter lacks a TATA box and contains an initiator element (Inr) necessary for its transcription. Like most TATA-less promoters, *ABCB1* promoter contains an inverted CCAAT box (Y box) close to a −50GC box. These two elements, where NF-Y, Sp1, and Sp3 factors can bind, form an enhancesome and recruit the histone acetyltransferase (HAT) PCAF. This HAT further acetylates histones at the proximal promoter region and then induces transcriptional activation of *ABCB1* gene. Several other elements have also been described among which a −110GC box able to bind a repressor factor. Accessibility of *ABCB1* promoter elements to their binding factors is regulated at the chromatin level by epigenetic regulatory mechanisms including DNA methylation and histones post-translational modifications. Among these modifications, acetylation of lysine residues of histone tails by HAT has been correlated with transcriptional activity, whereas deacetylation mediated by histone deacetylases (HDACs) has been associated with gene silencing (Grunstein, 1997; Berger, 2002).

Histone deacetylase inhibitors have been shown to have anti-tumour effects and a number of them are currently being evaluated in cancer therapy, either alone or in combination with conventional cytotoxic therapy (Yoo and Jones, 2006). In this regard, the impact of HDAC inhibition on *ABCB1* gene expression seems to be of critical importance.

We and others showed previously that HDAC inhibition induced similar proliferation inhibition and apoptotic response in H69 drug-sensitive and drug-resistant cells. This was associated with a blockade of the cells in the G_0_/G_1_ and the G_2_/M phases of the cycle in both cell lines (Tsurutani *et al*, 2003; El-Khoury *et al*, 2004). However, this inhibition induced very different morphological consequences at the chromatin condensation level in drug-sensitive and drug-resistant cells (El-Khoury *et al*, 2004). Such data could raise questions on the effects of HDAC inhibition on *ABCB1* expression in these cell variants.

In this study, we have utilised the HDAC inhibitor, trichostatin A (TSA), to investigate the responsiveness of endogenous *ABCB1* gene to acetylation states in SCLC H69 cells, either sensitive (H69WT) or resistant to etoposide (H69VP). This response was analysed in terms of dependence on transcription, DNA methylation, or histone acetylation at the promoter level. Moreover, the role of transcription regulators as PCAF and MBD1 was also evaluated.

## MATERIALS AND METHODS

### Chemicals

The HDAC inhibitors TSA and sodium butyrate (NaB), actinomycin D (ActD), and cycloheximide (CHX) were purchased from Sigma-Aldrich (St Quentin-Fallavier, France). All antibodies against histones and peroxidase-conjugated anti-rabbit secondary antibody were from Upstate Biotechnology (Lake Placid, NY). Phycoerythrin-conjugated antibody against P-gp (15D3-PE) was purchased from BD Biosciences (Grenoble, France), anti-PCAF antibody from Santa Cruz Biotechnology (Santa Cruz, CA, USA), and anti-MBD1 antibody from Abcam (Cambridge, UK). All other chemicals were obtained from standard sources.

### Cells

The human small cell lung carcinoma (SCLC) cell line H69 was obtained from ATCC (HTB-119) and its P-gp-positive multidrug-resistant variant H69VP selected with etoposide was provided by Professor Maxwell Sehested (Rigshospitalet, Copenhagen, Denmark) (Brock *et al*, 1995). The two cell lines were grown as previously reported (El-Khoury *et al*, 2004). The H69VP cells are about 17-fold resistant to etoposide and 8-fold and 6-fold cross-resistant to vincristine and doxorubicin, respectively.

### Anti-P-gp immunostaining and fluorescence-activated cell sorting (FACS) analysis

Cells were stained with phycoerythrin-conjugated anti-P-gp monoclonal antibody 15D3-PE according to the manufacturer's instructions. A total of 2 × 10^4^ cells were immediately analysed on a FACSCalibur flow cytometer (Becton Dickinson, San Jose, CA, USA) with an argon laser set at 488 nm. Data were analysed with BD Cell Quest software.

### Real-time and classical reverse transcription (RT)–PCR

Total cellular RNA was prepared using the guanidine thiocyanate/phenol method (Tri Reagent, Sigma). RNA (1 *μ*g) was reverse transcribed into cDNA in a final volume of 20 *μ*l using the ImProm-II Reverse Transcription System Kit (Promega, WS). Real-time PCR was performed with QuantiTectSYBR-Green PCR Kit (Qiagen, Courtaboeuf, France) on the LightCycler system (Roche Diagnostics, Basel, Switzerland) following the recommendation of the manufacturer. The expression levels of *ABCB1* mRNA were normalised against 18S ribosomal RNA level of the same sample. To detect *WTH3* gene expression, classical RT–PCR was carried out using recombinant Taq polymerase (Invitrogen, France) on a PCR System Thermal Cycler (Eppendorf, France) and its expression level was normalised against glyceraldehyde-3-phosphate dehydrogenase (*GAPDH*) mRNA. PCR primers and conditions are available upon request. Values reported represent the mean gene expression from at least three separate experiments±s.e.m. Data were analysed using Student's *t*-test.

### Acid extraction and immunoblotting of histones

Histones acid extraction and immunoblotting analysis were performed as described previously (El-Khoury *et al*, 2004). Antibodies were used at the following dilutions: anti-histone H3 (1 : 2000), anti-acetylated histone H3 (1 : 20 000), anti-acetylated histone H4 (1 : 2000), and anti-dimethylated histone H3 on lysine 9 (H3K9) (1 : 2000), peroxidase-conjugated anti-rabbit secondary antibody (1 : 2000).

### Acetic acid-urea-triton (AUT) gel electrophoresis

Acetic acid-urea-triton gel electrophoresis of histones was carried out following the protocol described previously (El-Khoury *et al*, 2004).

### Chromatin immunoprecipitation (ChIP) assay

The ChIP assay kit from Upstate Biotechnology was used following the manufacturer's instructions with the following modifications: cross-linking with formaldehyde was carried out for 10–20 min. Chromatin was immunoprecipitated with 8 *μ*g of either anti-acetylated histone H3, anti-acetylated histone H4, anti-dimethylated H3K9, anti-PCAF, or with 15 *μ*l of anti-MBD1 antibody. Three regions of the *ABCB1* promoter were analysed: a proximal region (+292 to +591), a distal region (−981 to –817), and the inverted CCAAT box (Y box) region (−222 to +37). In the case of histones and PCAF analyses, PCR products were resolved on 2% agarose gels stained with ethidium bromide, and quantitated using Typhoon 9210 scanner and Image Quant analysis software (Amersham Biosciences, Orsay, France). For MBD1 analysis, real-time PCR was carried out. PCR primers and conditions are available upon request.

### Bisulphite treatment of genomic DNA and methylation-specific PCR

Genomic DNA was isolated using NucleoSpin Tissue kit (Macherey-Nagel, Hoerdt, France) according to manufacturer's protocol. A total of 2 *μ*g of genomic DNA was denatured in 0.3 mol l^−1^ NaOH for 15 min at 37°C. The denaturated DNA was incubated with 0.5 mM hydroquinone and 3.1 mol l^−1^ sodium bisulphite (pH 5) for 16 h at 50°C. DNA was purified using the Wizard DNA clean-up system (Promega) and desulphonated with 0.3 N NaOH at 37°C for 15 min. DNA was precipitated with ammonium acetate and ethanol, washed with 70% ethanol, and resuspended in 20 *μ*l distilled water. For methylation-specific PCR analysis, the bisulphite-modified DNA samples were amplified by primers specific for both methylated and unmethylated sequences of the −50GC and −110GC boxes of the *ABCB1* promoter. DNA amounts were demonstrated in a PCR analysis using a primer set designed to amplify both the bisulphite-modified methylated and unmethylated sequences of the region including −50GC and −110GC boxes. PCR primers and conditions are available upon request. PCR products were analysed in 2% agarose gel stained with ethidium bromide.

### Combined bisulphite restriction analysis (COBRA)

This method enables a quantitative analysis of methylation at specific gene loci (Xiong and Laird, 1997). Bisulphite-modified genomic DNA was amplified by PCR. PCR primers sequences of the Inr region (−20 to +172) and PCR conditions are available upon request. Purified PCR products (192 bp) were digested with restriction enzyme *Taq*I (Invitrogen), which recognises the TCGA palindromic sequence unique to the bisulphite-converted DNA of the methylated alleles of the Inr site. DNA samples were precipitated and electrophoresed in 2.5% agarose gel stained with ethidium bromide. The intensity of methylated alleles was calculated by densitometry using Typhoon 9210 scanner and Image Quant analysis software (Amersham Biosciences).

## RESULTS

### TSA and NaB induce *ABCB1* upregulation in H69WT-sensitive cells but downregulation in H69VP-resistant cells

**Figure 1:**
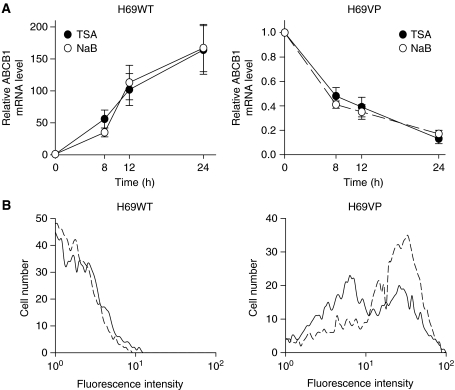
Trichostatin A (TSA) effects on *ABCB1* gene in H69 cells. (**A**) *ABCB1* expression in H69WT (left) and H69VP (right) cells treated with TSA (330 nmol l^−1^) or sodium butyrate (NaB, 5 mmol l^−1^) for 8, 12, and 24 h. (**B**) Decrease in P-gp membrane expression in H69VP cells treated with TSA. Fluorescence-activated cell sorting analysis was performed with 15D3-PE antibody on H69WT and H69VP cells treated or not with TSA. Dashed line, control cells; solid line, TSA-treated cells. *ABCB1* expression was normalised against 18S RNA expression (**A**). Quantitation of the relative changes in gene expression was measured by real time RT–PCR (mean±s.e.m. of 3–6 independent experiments).

Several studies indicate that *ABCB1* gene activity is regulated by HDACs. To analyse this regulation in drug-sensitive and drug-resistant cells, H69WT and H69VP cells were exposed to the HDAC inhibitors TSA and NaB (330 nmol l^−1^ and 5 mmol l^−1^ concentrations, respectively) for up to 24 h. These concentrations were shown previously optimal for inhibition of HDACs with minimal toxicity in these particular cells (El-Khoury *et al*, 2004). Trichostatin A and NaB induce significant increases in *ABCB1* expression in H69WT cells whose basal expression was extremely low (Figure 1A, left). This expression appears as early as 8 h of treatment with both drugs (56-fold increase with TSA, *P*=0.0005; 35-fold increase with NaB, *P*=0.008) and increases after 24 h of treatment (164-fold increase with TSA, *P*=0.008; 167-fold increase with NaB, *P*=0.008). On the contrary, TSA and NaB induce a very significant decrease in *ABCB1* expression in H69VP drug-resistant cells (Figure 1A, right). Significant changes can be noted after 8 h of treatment (52% inhibition with TSA, *P*<0.0001; 59% inhibition with NaB, *P*=0.0003) and 24 h of treatment with both drugs (87% inhibition with TSA, *P*<0.0001; 83% inhibition with NaB, *P*=0.0001). P-glycoprotein expression on cell membranes was analysed by flow cytometry on TSA-treated cells. H69WT and H69VP cells were incubated with or without 330 nmol l^−1^ TSA for 12 h and left for a further 48 h period in TSA-free medium. This TSA treatment does not result in significant changes in P-gp expression in H69WT cells (98% negative cells in TSA-treated or -untreated cells), whereas a decrease in this expression is observed in H69VP cells (Figure 1B), either when data are expressed as percent of P-gp-positive cells (76% +ve cells in control cells, 55% +ve cells in the presence of TSA) or as mean fluorescence index in these positive cells (39 *vs* 29 respectively), suggesting that *ABCB1* gene silencing observed in these drug-resistant cells after TSA treatment could effectively result in inhibition of P-gp expression at the cell membrane level.

### TSA-induced *ABCB1* modulation occurs at a transcriptional level

**Figure 2:**
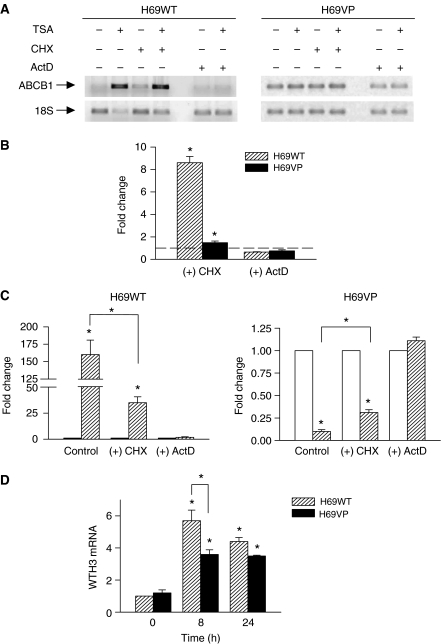
*ABCB1* up- or downregulation by trichostatin A (TSA) occurs through a transcription-dependent mechanism. (**A**) Expression of *ABCB1* in cells in presence of TSA, cycloheximide (CHX), or actinomycin D (ActD). Cells were pretreated with CHX (10 *μ*g ml^−1^, 1 h) or ActD (5 *μ*g ml^−1^, 30 min) before incubation in the presence or absence of TSA (330 nmol l^−1^, 24 h). PCR reactions were interrupted in the exponential phase and PCR products were electrophoresed in 2.5% agarose gel stained with ethidium bromide. (**B**) Expression of *ABCB1* in cells treated with CHX or ActD alone. H69WT and H69VP cells were incubated in presence or absence of CHX (10 *μ*g ml^−1^, 1 h) or ActD (5 *μ*g ml^−1^, 30 min). (**C**) Quantitation of *ABCB1* expression changes in cells treated with TSA (hatched bars) or not (open bars) in the presence or absence of CHX or ActD. Cells were treated as in (**A**). (**D**) *WTH3* expression in H69 cells treated with TSA (330 nmol l^−1^). *ABCB1* expression was normalised against 18S RNA expression. Quantitation of the relative changes in *ABCB1* expression was measured by real time RT–PCR. *WTH3* expression was normalised against *GAPDH* RNA expression. Relative changes in *WTH3* expression were measured by classical RT–PCR (mean±s.e.m. of three independent experiments). ^*^P<0.05.

**Figure 3:**
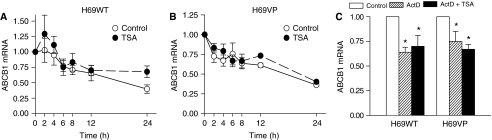
*ABCB1* up- or downregulation by trichostatin A (TSA) is not mediated by mRNA stability changes. (**A**) Analysis of a transcription-independent TSA effect on *ABCB1* mRNA stability in H69WT cells. Cells were treated with 5 *μ*g ml^−1^ actinomycin D (ActD) 30 min before the addition of 330 nmol l^−1^ TSA. Cells were harvested immediately or as indicated. (**B**) Analysis of a transcription-independent TSA effect on *ABCB1* mRNA stability in H69VP cells. Cells were treated and harvested as in (**A**). (**C**) Analysis of a transcription-dependent TSA effect on *ABCB1* mRNA stability. H69WT and H69VP cells were pretreated with 330 nmol l^−1^ TSA for 6 h before the addition of ActD (5 *μ*g ml^−1^, 30 min). Cells were harvested immediately or 12 h later for *ABCB1* mRNA quantitation. *ABCB1* expression was normalised against 18S RNA expression. Quantitation of the relative changes in *ABCB1* expression was measured by real time RT–PCR (mean±s.e.m. of three independent experiments). ^*^P<0.05.

To investigate the mechanisms implicated in these up- or downregulations, we first addressed the question whether ABCB1 expression is directly regulated by TSA in H69 cells. To this end, H69WT and H69VP cells were pretreated for 1 h with the protein synthesis inhibitor CHX at 10 *μ*g ml^−1^ followed or not by treatment with 330 nmol l^−1^ TSA for 24 h. Cycloheximide alone increased *ABCB1* mRNA levels in both H69WT and H69VP cells (7-fold increase, *P*=0.002, and 1.5-fold increase, *P*=0.02, respectively) (Figure 2A and B). When TSA was added, *ABCB1* mRNA modulation still occurred in both H69WT and H69VP cells in the presence of CHX. However, CHX induced a partial but significant inhibition of these modulations (Figure 2C). Thus, *de novo* protein synthesis enhances but is not responsible for TSA effect on *ABCB1* expression. These results suggest that TSA modulates *ABCB1* expression by a direct effect on chromatin, possibly by promoting recruitment of a repressor factor to *ABCB1* promoter, as well as through the modulation of regulatory proteins expression. Among these, WTH3 was reported to play a negative role in *ABCB1* gene expression. Moreover, *WTH3* gene expression seemed to be regulated by epigenetic mechanisms (Tian *et al*, 2005). However, TSA induced an overexpression of *WTH3* in both cell lines with a more important increase in drug-sensitive cells where *ABCB1* expression was upregulated (Figure 2D). These results suggest that WTH3 was probably not a key element in TSA-induced *ABCB1* inhibition in H69VP cells. To analyse the effects of TSA on *ABCB1* transcription, H69WT and H69VP cells were incubated with the RNA synthesis inhibitor ActD at 5 *μ*g ml^−1^ for 30 min before TSA treatment (330 nmol l^−1^ for 24 h). Pre-incubation with ActD completely inhibited TSA effects on *ABCB1* expression in H69WT and H69VP cells (Figure 2A and C). These data suggest that TSA regulates *ABCB1* expression at the transcriptional level. However, regulation of mRNA abundance can also occur at the level of RNA degradation. To examine if modifications of RNA stability could also account for TSA effects on *ABCB1* mRNA levels, H69WT and H69VP cells were pretreated with ActD at 5 *μ*g ml^−1^ to block new RNA synthesis. Then, the cells were treated or not with 330 nmol l^−1^ TSA. *ABCB1* mRNA half-lives appeared almost identical in both H69WT and H69VP cells, and TSA treatment did not significantly change these mRNA stabilities (Figure 3A and B). Finally, we examined if TSA could change *ABCB1* mRNA degradation through transcriptional modulation of other genes implicated in mRNA stability. For this purpose, H69WT and H69VP cells were incubated with or without 330 nmol l^−1^ TSA for 6 h before ActD treatment for 30 min at 5 *μ*g ml^−1^. *ABCB1* mRNA was then analysed immediately and 12 h later. *ABCB1* mRNA level decreased of about 40% after 12 h in H69WT cells, whether or not they were pre-incubated with TSA. Similar decreases of about 30% were observed in H69VP cells (Figure 3C). Thus, TSA modulates *ABCB1* mRNA transcription both in H69WT and H69VP cells without influencing its degradation.

### TSA increases the overall acetylation of histones but differentially modulates H3 and H4 acetylation levels at the *ABCB1* promoter level in sensitive and resistant cells

**Figure 4:**
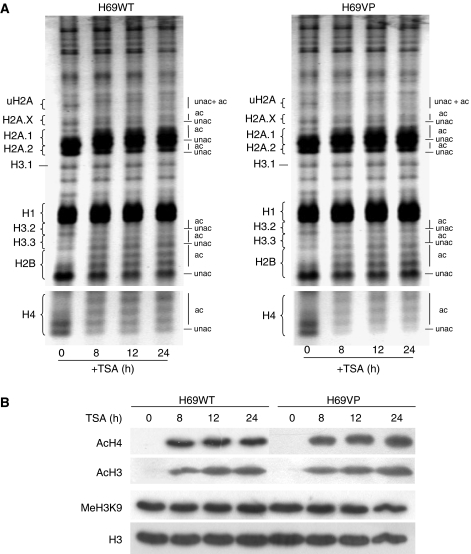
Trichostatin A (TSA) induces global histone acetylation. (**A**) Acetic acid-urea-triton-polyacrylamide gel electrophoresis of histones extracted from H69WT and H69VP nuclei. (**B**) Western blot analysis of acetylated H4, acetylated H3, and dimethylated H3K9 levels in nuclear acid extracts from H69WT and H69VP cells. Total histone H3 was used as a loading control.

**Figure 5:**
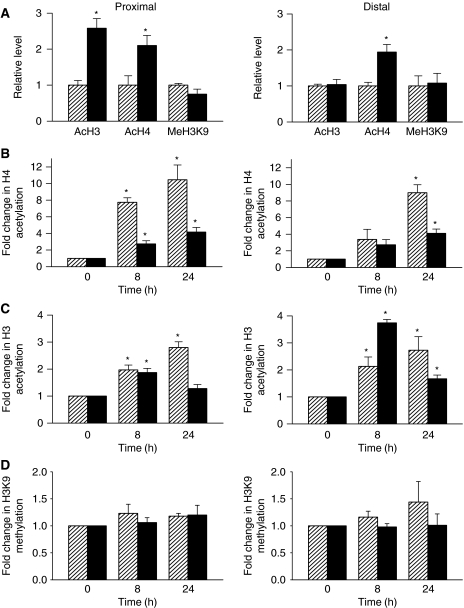
Trichostatin A induces differential temporal histone acetylation at the *ABCB1* promoter level. (**A**) Relative level of H3 acetylation, H4 acetylation, and H3K9 dimethylation at the proximal and distal regions of the *ABCB1* promoter in untreated H69WT (hatched bars) and H69VP cells (black bars). Fold change in acetylated H4 (**B**), acetylated H3 (**C**), and dimethylated H3K9 (**D**) at the proximal and distal regions of the *ABCB1* promoter in H69WT (hatched bars) and H69VP (black bars) cells treated with TSA (330 nmol l^−1^, 8, and 24 h). PCR amplifications of ChIP samples (mean±s.e.m. from three independent experiments). ^*^P<0.05.

The biological consequence of HDACs inhibition is the accumulation of acetylated histones. To verify the activity of TSA in our study, H69WT and H69VP cells were treated with 330 nmol l^−1^ TSA for up to 24 h, and histones were analysed by AUT-polyacrylamide gel electrophoresis (PAGE) (Figure 4A). The main changes observed, both in H69WT and H69VP cells, were an increase in H4 and H3 acetylation, particularly at the H3.3 and H3.2 levels, together with an increase in H2A and H2B acetylation. On the contrary, H1 remains unaffected by TSA, as this histone form cannot be acetylated through post-translational processes *in vivo* (Alvelo-Ceron *et al*, 2000). To confirm these data, the acetylated histone H3, acetylated histone H4 and methylated H3K9 levels were determined by western blotting. Consistent with previous observations, we found that TSA increased the acetylated histone H3 and H4 without any change in methylated H3K9 in both H69WT and H69VP cells (Figure 4B). To further analyse the implications of these histone acetylation changes on *ABCB1* expression, we performed ChIP assays to determine histone acetylation within specific regions of the *ABCB1* promoter. Primer sets encompassing *ABCB1* proximal (+292 to +591) and distal (−981 to −817) promoter regions were used to map changes in histone acetylation following TSA treatment. In comparison with H69WT drug-sensitive cells in which the *ABCB1* gene is underexpressed, H69VP drug-resistant cells had increased H4 acetylation in both regions (about twofold) and increased H3 acetylation (about 2.5-fold) in the proximal region of the promoter. H3K9 methylation levels appeared similar in H69WT and H69VP cells (Figure 5A). Following TSA treatment at 330 nmol l^−1^ for up to 24 h, H4 acetylation progressively increased at both promoter regions in the two cell lines (Figure 5B). The increase in H4 acetylation levels was lower in the resistant cells, probably due to the basal hyperacetylation of histone H4 in these cells at both promoter regions (Figure 5A). An increased H3 acetylation was also observed in H69WT cells. However, in H69VP cells, TSA induced an only transient H3 hyperacetylation, with a 2- to 4-fold increase after 8 h treatment and a return towards basal levels after 24 h (Figure 5C). If we consider the fold difference in basal H3 acetylation between drug-sensitive and drug-resistant cells (about 2.5-fold in the proximal region and no difference in the distal region), we can notice that histones H3 in H69VP drug-resistant cells are approximately twice as hyperacetylated as in H69WT drug-sensitive cells after 8 h treatment with TSA. The histone H3 deacetylation after 24 h treatment was not observed by western blotting of total nuclear histone H3 (Figure 4B), suggesting that this phenomenon remains localised. Trichostatin A treatment did not modify H3K9 methylation status at both promoter sites in H69WT or H69VP cells (Figure 5D). These results show that, as expected, the transcriptional activation of *ABCB1* induced by TSA in H69WT drug-sensitive cells was associated with hyperacetylation of histones H3 and H4 at *ABCB1* promoter. On the contrary, hyperacetylation of histones H3 and H4 observed in H69VP drug-resistant cells after 8 h treatment with TSA was associated with *ABCB1* gene silencing (Figure 1A).

### *ABCB1* repression by TSA appears independent of DNA methylation status and MBD1 recruitment in H69VP cells

**Figure 6:**
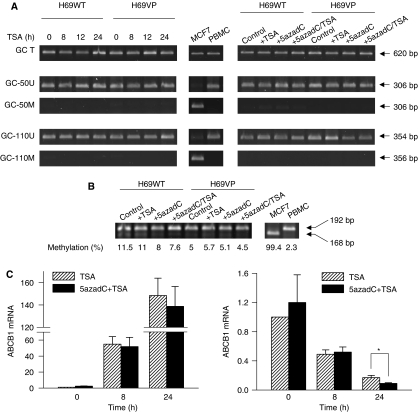
Trichostatin A (TSA)-induced up- and downregulations of *ABCB1* expression appear independent of *ABCB1* promoter methylation. (**A**) Methylation-specific PCR of *ABCB1* −50GC and −110GC boxes. Primer sets are designed to amplify methylated (M) and unmethylated (U) alleles. A primer set encompassing both M and U whole GC region (T) was used as loading control. Left lane, MSP analysis in H69WT and H69VP cells treated with TSA for up to 24 h. Right lane, MSP analysis in H69WT and H69VP cells treated with TSA for 8 h, 5azadC for 72 h, or combination of both. MCF7 cells and peripheral blood mononuclear cells (PBMC) were used as positive controls for methylated and unmethylated alleles, respectively (central lane). Representative experiment from a series of three. (**B**) Combined bisulphite restriction analysis of the Inr *ABCB1* promoter region in H69WT and H69VP cells treated with TSA, 5azadC, or a combination of both. Figures represent the methylation percentages observed in the different samples (mean of two separate independent experiments). MCF7 cells and PBMC were used as positive controls for methylated and unmethylated Inr site, respectively. (**C**) *ABCB1* mRNA expression in H69WT (left) and H69VP (right) cells treated with TSA alone or in combination with 5azadC. *ABCB1* expression was normalised against 18S RNA expression. Quantitation of the relative changes in *ABCB1* expression was measured by real time RT–PCR (mean±s.e.m. of three independent experiments). ^*^P<0.05.

**Figure 7:**
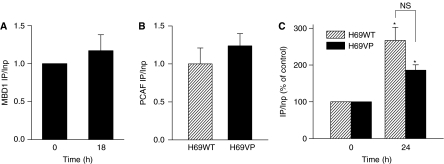
Trichostatin A (TSA)-induced inhibition of *ABCB1* expression in H69VP cells appears independent of MBD1 and PCAF binding to the *ABCB1* promoter CCAAT inverted box. (**A**) Chromatin immunoprecipitation analysis of MBD1 binding in H69VP cells treated with TSA (330 nmol l^−1^) for 18 h. (**B**) Chromatin immunoprecipitation analysis of PCAF binding in untreated H69WT and H69VP cells. (**C**) Relative changes in PCAF binding in H69WT and H69VP cells treated with TSA (330 nmol l^−1^) for 24 h. ^*^Significantly different from untreated controls. *P*<0.05 (mean±s.e.m. from three independent experiments).

*ABCB1* expression has been shown to be regulated not only by histone acetylation, but also by DNA methylation. *ABCB1* promoter contains several GC boxes, which appear essential for its activation. Among these, the −50GC and −110GC boxes appear highly relevant as they seem to be binding sites for *ABCB1* activators and repressors. The methylation profiles of these two boxes have been analysed by methylation-specific PCR in H69WT and H69VP cells and compared with those from hypermethylated (MCF7 cells) or hypomethylated (peripheral blood mononuclear cells, PBMCs) controls. The −50GC and −110GC boxes appeared hypomethylated in both cell lines (Figure 6A). This hypomethylation of *ABCB1* promoter was confirmed by a COBRA analysis of the +4 cytosine-phosphate-guanine (CpG) site of the Inr region. Both cell lines appeared to display hypomethylated *ABCB1* DNA (Figure 6B). These data confirm the sensitivity of hypomethylated *ABCB1* promoter to the direct action of HDACs (El-Osta *et al*, 2002). Treatment of H69WT and H69VP by TSA (330 nmol l^−1^, 24 h), or the demethylating agent 5azadC (2 *μ*mol l^−1^, 72 h), or a combination of both (cells treated with 5azadC for 72 h were resuspended in fresh supplemented medium and harvested 33 h later. Trichostatin A treatment was applied during the last 8 or 24 h), did not influence significantly methylation status of these various promoter sites (Figure 6A and B), indicating that *ABCB1* gene modulation, induced by TSA in both cell lines, does not result from alterations in the methylation status of the *ABCB1* promoter. Finally, although treatment with 5azadC alone induced a significant increase in *ABCB1* expression in H69WT cells (about 2.5-fold increase, data not shown), it did not modulate TSA-induced upregulation of *ABCB1* expression in H69WT. Similarly, in the H69VP drug-resistant cells, the combined treatment with TSA and 5azadC did not enhance the level of *ABCB1* downregulation, except for 24 h treatment with TSA (Figure 6C), probably in relation with the activation of a repressor of *ABCB1* transcription. Thus, the low level of *ABCB1* promoter methylation we observed in H69WT and H69VP cells, or other methylated sequences outside GC boxes and Inr element analysed, do not appear critical for TSA action on *ABCB1* mRNA expression in these cells containing originally a hypomethylated *ABCB1* promoter. It has been reported that MBD1, a protein associated with methylation-dependent repression, could also bind non-methylated DNA (Baker and El Osta, 2004). Thus, MBD1 binding to the inverted CCAAT box (−79 to −75) of the *ABCB1* promoter was evaluated in H69VP cells by ChIP using an anti-MBD1 antibody. Trichostatin A treatment (330 nmol l^−1^, 18 h) did not induce any significant change in MBD1 recruitment on the promoter site (Figure 7A), suggesting that the *ABCB1* expression decrease observed in H69VP cells was not linked to a MBD1-mediated methylation-independent silencing.

### TSA treatment results in similar PCAF binding to *ABCB1* promoter CCAAT inverted box in both cell lines

Histone deacetylases inhibitors induce *ABCB1* transcription through the association of the NF-Y factor with the CCAAT inverted box and the PCAF HAT recruitment (Friedman *et al*, 2002). To determine the role of this process in the *ABCB1* regulations induced by TSA in H69 cells, the chromatin from H69WT and H69VP cells, treated or not with 330 nmol/LTSA for 24 h, has been immunoprecipitated by anti-PCAF antibody. The immunoprecipitated DNA was analysed by PCR using primers corresponding to the inverted CCAAT box promoter region. Before TSA treatment, PCAF binding to the *ABCB1* promoter appeared similar in H69WT and H69VP cells (Figure 7B). This suggests that basal *ABCB1* overexpression observed in H69VP cells does not result from an increased PCAF protein binding. Incubation of cells with TSA for 24 h resulted in an increase in PCAF recruitment in both cell lines (2- to 3-fold increase) without any significant difference between H69WT and H69VP cells response (Figure 7C). Then PCAF recruitment occurred at the *ABCB1* promoter level during TSA treatment, whether this treatment resulted in an up- or downregulation of the corresponding gene.

## DISCUSSION

Epigenetic mechanisms (histone acetylation, DNA methylation) have been shown to play a pivotal role in *ABCB1* gene expression in several tumour cell systems (El-Osta *et al*, 2002; Labialle *et al*, 2002; Scotto, 2003).

Results presented here show that HDAC inhibitors TSA and NaB increased *ABCB1* gene expression in H69WT drug-sensitive cells, but strongly inhibited its expression in H69VP drug-resistant cells at both the mRNA and protein levels. H69 cells constitutively express the ABCC1 drug transporter, with an overexpression in H69VP cells. *ABCC1* TATA-less gene promoter shares common regulatory elements with *ABCB1* (eg GC boxes, Sp1 binding site, putative AP-1 site). Interestingly, TSA also downregulated this *ABCC1* gene expression in H69VP drug-resistant cells (data not shown). Thus, our results support the idea that HDAC inhibitors could modulate MDR through simultaneous inhibition of different ABC transporters as recently suggested for 4-phenylbutyrate (Ammerpohl *et al*, 2007).

Simultaneous treatments with the transcription inhibitor ActD suggested that these up- and downregulations occurred at the transcriptional level without any change in mRNA stability as reported in leukaemic cells (Yague *et al*, 2003). *De novo* protein synthesis inhibition with CHX did not suppress TSA-mediated *ABCB1* modulations, suggesting that TSA influence mainly this gene transcription directly at the chromatin level. However, as CHX reduced the intensity of these changes, a modulation by TSA of the transcription of other genes implicated in *ABCB1* regulation could occur as well. Among these, WTH3 was shown to be upregulated by TSA in MCF7/Adr cells resulting in an inhibition of *MDR1* expression (Tian *et al*, 2005). Despite a similar increase in *WTH3* expression in H69VP cells, this factor may not be a good candidate for *ABCB1* inhibition by TSA as *WTH3* is also upregulated in TSA-treated H69WT cells exhibiting an increase in *ABCB1* expression. These conflicting results might be related to different methylation status observed in H69 or MCF7 cells (David *et al*, 2004). Cycloheximide alone appeared able to increase the basal *ABCB1* expression in H69 cells. This increase, which has already been described for other genes (Keller and Kneissel, 2005), could suggest that *ABCB1* expression would be controlled by short-lived repressor, or that CHX might directly stimulate *ABCB1* gene transcription as reported for *α*-1B adrenergic gene (Hu and Hoffman, 1993). In drug-sensitive cells, several studies have reported an increase in *ABCB1* expression by HDAC inhibitors, a phenomenon we also observed in H69WT cells. For instance, *ABCB1* gene was overexpressed in SW620 colon carcinoma and CEM-Bcl2 cells exposed to TSA (Jin and Scotto, 1998; Baker *et al*, 2005), or KU812 and NB4 cells exposed to depsipeptide (Tabe *et al*, 2006; Yamada *et al*, 2006). In drug-resistant cells, HDAC inhibition also increased *ABCB1* expression in CEM-A7R, Kasumi-1, and Kasumi-6 cell lines (El-Osta *et al*, 2002; Tabe *et al*, 2006). On the contrary, our results showed a repression of *ABCB1* gene expression by TSA and NaB. Direct inhibition of *ABCB1* expression by HDAC inhibitors in drug-resistant cells appeared as a relatively uncommon event, previously reported in murine L1210R cells (Castro-Galache *et al*, 2003).

To better understand the mechanisms of *ABCB1* regulation in H69 cells, we have investigated the effect of HDAC inhibition by TSA on histone post-translational modifications, focusing on H3 and H4 acetylation, and H3K9 methylation. We found that even though TSA increased global accumulation of acetylated histones, H3 and H4 acetylation displayed different temporal patterns at the *ABCB1* promoter level. Histone deacetylases inhibitors increase H3 and/or H4 acetylation at the promoter of activated genes such as T*β*RII (Zhao *et al*, 2003), IAP (Hinnebusch *et al*, 2003), or Mn-SOD (Maehara *et al*, 2002). We observed the same phenomenon in H69WT drug-sensitive cells both at distal and proximal regions of the *ABCB1* promoter. Examining the downstream +292 to +591 region of the gene give data on the histone modification of the transcribed part of the gene and therefore on possible regulation of elongation by histone modification. Concerning the −981 to −817 region, the T-cell factor-4 (TCF4)/*β*-catenin complex has been reported as an *ABCB1* transcriptional activator which binds seven elements spanning the −1813 to −261 sequence of the *ABCB1* promoter. The −981 to −817 region harbours one of these seven TCF elements (Yamada *et al*, 2000). It has been recently suggested that TCF4 plays an important role in the carcinogenesis of lung cancer as demonstrated by its high expression in cancer samples (Li *et al*, 2005). Besides, overexpression of *β*-catenin reported in lung cancer and Wnt signalling pathway, which results in *β*-catenin stabilisation and activation, is found to be aberrantly activated in lung cancer (Mazieres *et al*, 2005). Hence, examining the acetylation status of the −981 to −817 region of the *ABCB1* promoter seemed interesting in the H69 lung carcinoma cell lines. Changes in H3 and H4 acetylation were rapid (within 8 h) and directly correlated with *ABCB1* induction. Similar data have been reported in NB4 cells incubated with FK228 (Tabe *et al*, 2006), or in CEM-Bcl2 cells treated with daunorubicin (Baker *et al*, 2005). In TSA-treated H69VP cells, H4 acetylation exhibited the same kinetics than in drug-sensitive cells, in spite of the repression of the *ABCB1* gene induced. Furthermore, H3 acetylation displayed a biphasic curve with a transient hyperacetylation followed by a decrease in this acetylation level. Interestingly, TSA-mediated decrease in *ABCB1* mRNA occurred rapidly after 8 h when both H3 and H4 displayed a hyperacetylated pattern at the gene promoter level. Similar gene inhibition by TSA in spite of H4 acetylation at the promoter level has already been described for *PU.1* gene (Laribee and Klemsz, 2001). Several hypotheses can be proposed to explain this unexpected phenomenon: a nucleosome displacement induced by histone acetylation could result in masking of critical DNA activation sites (Laribee and Klemsz, 2001), or the gene repression could be linked to the acetylation by TSA of non-histone proteins implicated in gene transcription (Wilson *et al*, 2002; Mulholland *et al*, 2003). In H69VP cells, after 24 h of TSA treatment, H3 acetylation decreases whereas H4 acetylation continues to increase. This H3 deacetylation cannot be explained by TSA degradation or efflux as global hyperacetylation remains high at the whole nucleus level. This decrease in acetylated H3 might be linked to nucleosomes loss instead of H3 deacetylation (Deckert and Struhl, 2001), but this seems rather unlikely as this loss would result in concomitant decrease in acetylated H4 level. Deacetylation following a transient hyperacetylation of H3 and/or H4 by TSA has already been described at the level of mouse mammary tumour virus and c-jun promoters (Thompson *et al*, 2001; Mulholland *et al*, 2003), and even *ABCB1* promoter in CEM-Bcl2 cells (Baker *et al*, 2005). Nevertheless, it should be noted that the H3 acetylation level observed after 24 h TSA in H69VP cells was always above the basal value measured in untreated cells. Thus, H3 acetylation decrease could help or maintain *ABCB1* silencing, but would probably not act as a starting event of the process.

In addition to histone post-translational modifications, DNA methylation is one of the mechanisms controlling *ABCB1* transcription with an inverse relationship between promoter CpG methylation and *ABCB1* expression (Kantharidis *et al*, 1997; Nakayama *et al*, 1998; El-Osta *et al*, 2002). However, as we observed promoter hypomethylation in H69WT cells, *ABCB1* repression in drug-sensitive cells seems independent of its promoter methylation, a phenomenon previously reported in SW620 cells (Jin and Scotto, 1998; Baker *et al*, 2005). In H69 cells, TSA induces up- or downregulation of *ABCB1* without any significant modification of its promoter methylation status, as already observed in CCRF-CEM cells (El-Osta *et al*, 2002). It has been reported that *ABCB1* activation by HDACs can only occur when its promoter is demethylated, either spontaneously or after treatment with demethylating agent 5azacytidine (El-Osta *et al*, 2002). In a similar way, in H69WT cells, TSA alone can induce *ABCB1* expression since its promoter appeared hypomethylated and simultaneous incubation with 5azadC did not potentiate gene expression, as observed in hypomethylated CEM-Bcl2 and SW620 cells (Baker *et al*, 2005). 5azadC alone induced moderate *ABCB1* expression suggesting either a possible further slight demethylation of an already hypomethylated *ABCB1* promoter (as observed by COBRA analysis of the Inr sequence), or the demethylation of the promoter of other gene(s) implicated in *ABCB1* activation. Our results in H69VP drug-resistant cells demonstrate that TSA could induce *ABCB1* silencing in a hypomethylated promoter environment. This effect could be linked to the induction of an increase in DNA methylation level as it has been reported that NaB was able to increase this level in several cell lines (DeHaan *et al*, 1986; Cosgrove and Cox, 1990). Nevertheless, our results showed that *ABCB1* repression by TSA in H69VP cells was not associated with a hypermethylation of its promoter at the analysed sites. Furthermore, it has been suggested that MBD1 protein could play a role in *ABCB1* methylation-independent silencing (Baker and El Osta, 2004). The lack of MBD1 recruitment following TSA treatment argues against this possibility in H69VP-resistant cells.

To further elucidate possible mechanisms of TSA-mediated *ABCB1* regulations in H69 cells, the ChIP assay was performed to determine if PCAF binding to *ABCB1* promoter differs between drug-sensitive and drug-resistant cells and if PCAF occupancy changes following HDAC inhibition by TSA in these two cell lines. This protein, which possesses a HAT activity, can therefore activate basal or induced expression of *ABCB1* gene (Hu *et al*, 2000; Friedman *et al*, 2002; Tanaka *et al*, 2003). Moreover, *ABCB1* promoter is activated by PCAF overexpression (Jin and Scotto, 1998), but a direct association of PCAF with *ABCB1* promoter *in vivo* has not been previously demonstrated. Our results show that PCAF is bound to the *ABCB1* promoter in a similar extent in drug-sensitive and drug-resistant cells and reveal similar increases in the level of bound PCAF in TSA-treated H69WT or H69VP cells. These results suggest that the levels of PCAF binding to *ABCB1* promoter *in vivo* do not correlate with gene expression level in H69 drug-sensitive and drug-resistant cells, unlike NF-Y binding in SSC cells (Okamura *et al*, 2004). The increase in PCAF binding upon TSA treatment in H69WT cells fits well with previous reports on the role of this protein in *ABCB1* induction. However, the silencing of *ABCB1* gene by TSA in H69VP cells was accompanied by a similar increase in PCAF recruitment to the promoter inverted CCAAT box region. This suggests that the *ABCB1* gene repression induced by TSA in drug-resistant cells occurs independently of PCAF binding.

**Figure 8:**
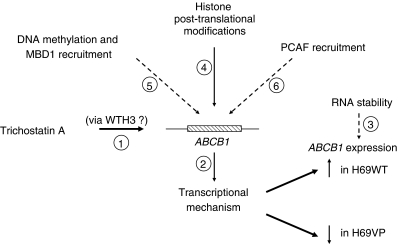
Pathways summary: trichostatin A modulates *ABCB1* gene expression in H69 cells (increase in H69WT cells but decrease in H69VP cells). This differential modulation occurs both through a direct action on chromatin and through changes in regulatory factors expression (1). Among these, WTH3 does not play a key role. This modulation occurs through transcriptional mechanisms (2) and is not linked to alterations in RNA stability (3). It is concomitant with differential histone acetylation patterns at the *ABCB1* promoter (4), but appears independent of DNA methylation and MBD1 (5) or PCAF recruitment (6) on CCAAT inverted box. Dashed lines represent pathways that cannot explain the differential regulation of *ABCB1* expression in H69WT and H69VP cells.

Taken together, our data provide evidence that HDAC inhibition can result in differential regulation of the *ABCB1* gene according to the resistance status of the SCLC H69 cells (Figure 8). Although a definite unique regulatory mechanism cannot be drawn from these results, they suggest that these regulations occur at a transcriptional level. The fact that *ABCB1* will be up- or downregulated in H69 cells appears independent of the methylation of the *ABCB1* −50GC, −110GC, and Inr promoter sites, and of MBD1 and PCAF binding, but is associated to different temporal patterns of histone acetylation at the *ABCB1* promoter level. Whereas the CCAAT inverted box plays a pivotal role in *ABCB1* upregulation by HDAC inhibitors, it seems that their repressive effect on this gene expression originates outside this element. Although TSA is not in clinical trials, this study supports the idea that HDAC inhibitors might be chemosensitising agents and promising drugs for combinatorial treatments with classical chemotherapeutic drugs. The characterisation by reporter constructs of the promoter sequences required for these up- and downregulations of *ABCB1* in H69 cells is currently under investigation in our laboratory to identify trans-regulatory factors mediating this differential TSA effect.

Most drug-resistant lung cancers overexpress both P-gp and MRP1 proteins (Triller *et al*, 2006). H69VP-resistant cells exhibit upregulation of both MDR proteins, somehow reflecting situations commonly seen in clinic. In this sense, the choice of this cell model seems appropriate. However, *ABCB1* promoter of H69VP cells is constitutively strongly hypomethylated, and the downregulation of *ABCB1* expression by HDAC inhibition could require such low level of DNA methylation in *ABCB1* promoter. Indeed, we have previously shown that *ABCB1* expression remains unchanged after TSA treatment of the multidrug-resistant human ovarian adenocarcinoma cell line OV1/VCR, in which *ABCB1* promoter is slightly hypermethylated compared to its sensitive IGROV1 counterpart (Yatouji *et al*, 2007). Further methylation analysis on different cell lines and clinical samples is required to clarify the relevancy of basal methylation status of *ABCB1* promoter on *ABCB1* repression by HDAC inhibitors.

Despite these limitations, this unique cellular model, which can display HDAC-mediated up- or downregulation of *ABCB1* gene according to resistance status, appears therefore useful for effective design of agents to reverse MDR in cancer patients.
